# Interaction of Arginine-Rich Cell-Penetrating Peptides with an Artificial Neuronal Membrane

**DOI:** 10.3390/cells11101638

**Published:** 2022-05-13

**Authors:** Piotr Mucha, Emilia Sikorska, Piotr Rekowski, Jarosław Ruczyński

**Affiliations:** 1 Laboratory of Chemistry of Biologically Active Compounds, Faculty of Chemistry, University of Gdańsk, Wita Stwosza 63, 80-308 Gdansk, Poland; piotr.rekowski@ug.edu.pl; 2 Environmental Nucleic Acid Laboratory, Faculty of Chemistry, University of Gdańsk, Wita Stwosza 63, 80-308 Gdansk, Poland; 3 Laboratory of Structural Research of Biopolymers, Faculty of Chemistry, University of Gdańsk, Wita Stwosza 63, 80-308 Gdansk, Poland; emilia.sikorska@ug.edu.pl

**Keywords:** cell-penetrating peptides, artificial neuronal membrane, liposome, molecular dynamics simulations, circular dichroism studies, PTD4 peptide, Tat(49–57)-NH_2_

## Abstract

Arginine-rich cell-penetrating peptides (RRCPPs) exhibit intrinsic neuroprotective effects on neurons injured by acute ischemic stroke. Conformational properties, interaction, and the ability to penetrate the neural membrane are critical for the neuroprotective effects of RRCCPs. In this study, we applied circular dichroism (CD) spectroscopy and coarse-grained molecular dynamics (CG MD) simulations to investigate the interactions of two RRCPPs, Tat(49–57)-NH_2_ (arginine-rich motif of Tat HIV-1 protein) and PTD4 (a less basic Ala-scan analog of the Tat peptide), with an artificial neuronal membrane (ANM). CD spectra showed that in an aqueous environment, such as phosphate-buffered saline, the peptides mostly adopted a random coil (PTD4) or a polyproline type II helical (Tat(49–57)-NH_2_) conformation. On the other hand, in the hydrophobic environment of the ANM liposomes, the peptides showed moderate conformational changes, especially around 200 nm, as indicated by CD curves. The changes induced by the liposomes were slightly more significant in the PTD4 peptide. However, the nature of the conformational changes could not be clearly defined. CG MD simulations showed that the peptides are quickly attracted to the neuronal lipid bilayer and bind preferentially to monosialotetrahexosylganglioside (DPG1) molecules. However, the peptides did not penetrate the membrane even at increasing concentrations. This suggests that the energy barrier required to break the strong peptide–lipid electrostatic interactions was not exceeded in the simulated models. The obtained results show a correlation between the potential of mean force parameter and a peptide’s cell membrane-penetrating ability and neuroprotective properties.

## 1. Introduction

Despite great progress in the medical field, many illnesses remain difficult to treat or are incurable, with cancers, harmful infections, and neurodegenerative disorders causing higher mortality. The therapies that are currently used for these diseases have many drawbacks, and clinicians strive to deal with them every day.

Many biologically active compounds, including both low-molecular-weight drugs and macromolecules, which are used as drugs to achieve a therapeutic effect, must be delivered to the interior of the cell or organelles such as mitochondria or nuclei. As the currently known drug delivery systems have certain limitations, there is a need to find new approaches that would allow for the effective transport of biologically active compounds into the cell interior. The efficient delivery of bioactive molecules (including therapeutic agents) to their targets is the key issue in modern medicine.

In recent years, cell-penetrating peptides (CPPs) have been receiving increased attention as cellular delivery vectors due to their ability to penetrate cell membranes as well as the blood–brain barrier [1,2,3,4,5]. CPPs are relatively short cationic peptides comprising approximately 30 amino acids. They can enter the cell and deliver different cargos, such as plasmids, DNA, siRNA, PNA, proteins, peptides, and low-molecular-weight drugs to their targets without causing any damage to the cells. Although the mechanisms by which CPPs penetrate the biological membranes remain unclear, it seems that they involve versatile endocytotic or nonendocytotic pathways. The mechanism of penetration of CPPs is determined by their chemical characteristics, molecular size of the cargo, and the type of cell they enter. CPPs, as a carrier of conjugates, are considered a fundamental part of a novel drug delivery system. However, some CPPs have been shown to possess potent neuroprotective properties themselves, which make them interesting candidates as therapeutic agents for various neurological disorders, including stroke and excitotoxicity-associated conditions [6].

Ischemic stroke (IS) is defined as a sudden disturbance of blood circulation in the brain, which results in the loss of function and the death of neurons [7]. It is one of the most dangerous and unsolved global health challenges to date. During the last decades, extensive efforts, unfortunately ineffective, have been made to identify drugs that can protect against IS [8,9]. Currently, there are no clinically effective neuroprotective drugs to protect neurons and brain tissue from IS-related degenerative effects. A recombinant serine protease tissue-type plasminogen activator that targets thrombus within a brain blood vessel is the only approved drug at present for the treatment of IS [10]. However, its pharmacological application has serious limitations.

Recently, arginine-rich cell-penetrating peptides (RRCPPs), such as poly-arginine, penetratin, or Tat(48–57) HIV-1, have been found to exhibit neuroprotective effects in in vitro neuronal cell stroke models [6,11,12,13], which may be a new possibility to design novel neuroprotective drugs. However, the mechanism underlying RRCPPs-dependent neuroprotection remains to be studied. It is known that RRCPPs are characterized by a multimodal mechanism of action, including the ability to protect neurons from glutamate excitotoxicity, reduce intracellular calcium influx, antagonize cell surface receptor function, target mitochondria, scavenge toxic molecules, reduce oxidative stress, induce prosurvival signaling, stabilize proteins, inhibit proteolytic enzymes, and reduce inflammation [6]. However, endocytosis seems to be a much more likely neuroprotective mechanism of RRCCPs rather than interaction with a membrane or cytoplasmic receptor [13].

Based on previous findings of the neuroprotective properties of RRCPPs, this study aimed to characterize the interaction of these peptides with an artificial neuronal membrane (ANM) mimicking liposomes as a model of a neuronal cell membrane. The study focused on gaining a better understanding of the mechanism of RRCPP–cell membrane interaction, which is responsible for the neuroprotective effects of the peptides. For this purpose, two RRCPP-class peptides—Tat(49–57)-NH_2_ (R^49^KKRRQRRR^57^-amide) and its less basic Ala-scan analog PTD4 (Y^47^ARAAARQARA^57^-amide, an analog of Tat protein fragment with 33-fold higher penetrating ability than the original peptide)—were used. The ANM composition used in this study was based on data presented previously [14]. Despite the fact that the lipid composition of synthetic models of neuronal membranes available in the literature is different, we believe that the one used in the research included in the publications of Fatafta et al. [14], as well as in our research, is the most complex in terms of the lipid composition and the closest to the membrane composition of the CNS neuron.

## 2. Materials and Methods

### 2.1. Reagents

All reagents and solvents were of analytical HPLC grade (Merck KGaA, Darmstadt, Germany). The solutions were freshly prepared in distilled deionized water using a Milli-Q Millipore system (Bedford, MA, USA) and filtered with a 0.22-μm filter before use. Fmoc (fluorenyl-9-methoxycarbonyl)-protected l-amino acids (Bachem AG, Bublendorf, Switzerland) and Rink-Amide TentaGel S RAM resin (Rapp Polymere GmbH, Tuebingen, Germany) were used for peptide synthesis. Various lipids (POPC, 1-palmitoyl-2-oleoyl-sn-glycero-3-phospho-choline; POPE, 1-palmitoyl-2-oleoyl-sn-glycero-3-phosphoethanolamine; POPS, 1-palmitoyl-2-oleoyl-sn-glycero-3-phospho-l-serine; CHOL, cholesterol; DPG1, monosialotetrahexosylganglioside; DPSM, sphingomyelin; all from Merck KGaA, Darmstadt, Germany) were used for liposome preparation.

### 2.2. Preparation of Liposomes

A defined amount of a mixture of lipids (POPC/POPE/POPS/CHOL/DPSM/DPG1, 38:24:5:20:9:4, expressed in percent mass ratio) was dissolved in chloroform, and the solution was gently dried under a continuous nitrogen flow and then overnight under high vacuum (1 mbar) to remove all remaining traces of the solvent [14,15]. The combined dried lipid mixture was resuspended in degassed 10 mM phosphate-buffered saline (PBS, pH 7.0) at room temperature and extensively vortexed for 5 min. The formation of ANM liposomes was induced by sonication at 75 °C using a Branson Sonifier 450. Sonication was carried out for 10 min with 1 min gap intervals. This was followed by five freeze–thaw cycles (freezing to −196 °C using liquid nitrogen and thawing at 65 °C for 5 min). Finally, the ANM liposomes with a diameter of 0.1 µm were formed by extrusion using an Avanti Mini-Extruder (Avanti Polar Lipids, Alabaster, AL, USA) through Nucleopore Track-Etched Polycarbonate 0.1 µm pore sized membrane (Whatman, Nucleopore polycarbonates, 20 cycles, Sigma Aldrich, Poznań, Poland). The resulting suspensions of ANM liposomes were incubated for 30 min at 37 °C to facilitate homogeneity, as confirmed by capillary electrophoresis analysis (data not shown). The final concentration of ANM liposomes was 1.25 mM.

### 2.3. Peptide Synthesis and Purification

CPPs were synthesized in an automated peptide synthesizer (Quartet, Protein Technologies Inc, Tucson, AZ, USA) as C-terminal amides on TentaGel S RAM amide resin (loading 0.25 mM/g) by applying Fmoc chemistry and TBTU (*O*-(benzotriazole-1-yl)-1,1,3,3-tetramethyluronium tetrafluoroborate) as a coupling reagent [16,17]. The synthesized peptides were cleaved from the peptidyl resin by treating them with a mixture of trifluoroacetic acid (TFA)/phenol/water/triisopropylsilane (88/5/5/2) at room temperature (RT) for 3 h under argon bubbles. The crude peptides were precipitated, washed with ice-cold diethyl ether, and then lyophilized. After lyophilization, the peptides were purified by a preparative reversed-phase high performance liquid chromatography HPLC (RP-HPLC) system (SpotPrep, Armen, Brittany, France) using a Reprosil 100 C-18 column (Dr. Maisch GmbH, 40 × 250 mm, 10 µm particle size). Several gradients of acetonitrile (ACN) with 0.08% TFA, at a flow rate of 25 mL/min, were used for purification. Fractions of the highest purity (>95%) were analyzed by an analytical RP-HPLC system (Prominence, Shimadzu, Duisburg, Germany) using a Phenomenex Kinetex XB-C18 column (150 mm × 4.6 mm, 5 µm particle size) with several gradients of ACN with 0.08% TFA at a flow rate of 1 mL/min. After purification and lyophilization, the peptides were dissolved in 0.01 M AcOH for trifluoroacetate-to-acetate ion exchange and then relyophilized. Finally, the homogeneity of the peptides was analyzed by analytical RP-HPLC (Prominence, Shimadzu) and mass spectrometry (TripleTOF 5600+, Sciex, Framingham, MA, USA).

### 2.4. CD Spectroscopy

Circular dichroism (CD) spectra of peptides were recorded on a Jasco J-815 spectropolarimeter (Jasco Int. Co., Ltd., Tokyo, Japan), at a peptide concentration of 0.15 mg/mL in 10 mM PBS buffer (pH 7.0) using 0.1 cm path length cuvettes. Measurements were taken at 37 °C in a spectral range of 195–250 nm with a bandwith of 1 nm and response of 1s. The temperature was controlled by a Peltier system built into the CD spectrophotometer with an increase of 1 °C per min. The concentration of the ANM liposomes was 1.25 mM and the lipid/peptide molar ratio was L/P = 10. The lyophilized peptide sample was dissolved in the ANM solution.

Spectra were recorded for both unbound and liposome-titrated peptides. The measurements were read straightaway after 30 min of titration of the peptides with the ANM liposomes. To exclude the interference of the liposome signal in the CD spectra of the peptide, the titrated spectra were subtracted from the CD signals of the liposomes, which were recorded at the same concentration in the absence of the peptide. Ellipticity was measured in mdeg units. The scan speed was 50 nm/min. Each CD spectrum was recorded six times and then averaged.

### 2.5. CG MD Simulations

The coarse-grained molecular dynamics (CG MD) simulations were carried out using the MARTINI force field [18,19] in the GROMACS 2019.5 package https://manual.gromacs.org/documentation/2019.5/ (accessed on 12 May 2022) [20]. The neuronal membrane model was comprised of 152 POPC, 96 POPE, 20 POPS, 80 CHOL, 36 DPSM, and 16 DPG1, which were equally distributed between two leaflets of the membrane, which was built in the CHARMM-GUI web-based graphical interface [21,22,23,24]. Membrane without water and counterions were used to construct the systems with Tat(49–57)-NH_2_ (RKKRRQRRR-NH_2_) and PTD4 (YARAAARQARA-NH_2_) peptides, with different concentrations (Table S1). The first system contained only a single peptide molecule, while others contained 10 peptide molecules randomly distributed above the outer membrane leaflet using the “insert-molecules” tool of the GROMACS package (after 1 µs of CG MD simulations, five additional peptide molecules with counterions were added to the systems). Each system was solvated with a standard MARTINI water model and neutralized with sodium and chloride counterions to keep the salt concentration for bulk solution at 150 mM NaCl. The systems were energy-minimized and equilibrated with stepwise-lowered force constant of harmonic restraints (from 2 to 0.1 kJ mol^−1^ Å^−2^), in order to fix the position of the headgroups of the membrane lipids and peptide during simulations. Finally, each system was subjected to isothermal-isobaric molecular dynamics (NPT) with a time step of 10 fs, as suggested by Wigner et al. [25]. In all simulations, lipids, peptide, and solvent were coupled separately at 310 K using the V-rescale thermostat. Pressure (1 bar) was regulated using a semi-isotropic Parinello-Rahman pressure coupling scheme. Coulomb interactions were treated using a reaction field and a cutoff of 11 Å, and the Lennard–Jones potential cut-off was set to 11 Å. Periodic boundary conditions were applied in all directions. A flat-bottom harmonic restraint was applied between each peptide molecule and membrane to prevent peptide molecules from drifting away from the outer membrane–water interface and binding to the inner leaflet due to periodic boundary conditions.

### 2.6. PMF Calculations

The free energy of Tat(49–57)-NH_2_ and PTD4 across the neuronal lipid bilayer was computed from the potential of mean force (PMF) parameter by umbrella sampling [26]. The bilayer normal was chosen as the reaction coordinate with *z* = 0 in the center of the membrane. The separation distance corresponds to the distance between the center of mass (COM) of the peptide and the lipid bilayer. In the initial configurations generated for umbrella sampling simulations, the peptide was placed at the *z*-axis distance in the range of 50–60 Å from the COM of the pre-equilibrated membrane. The system was neutralized by adding chloride counterions. The total salt concentration was maintained at 150 mM. The system was minimized and equilibrated as described in the Section 2.5. The peptide was pulled along the z-axis (perpendicular to the bilayer normal) from the bulky water at the outer side of the bilayer, through the bilayer center to the bulky water at the inner side of the bilayer, using the “direction” technique over 10 ns at a rate of 0.01 Å ps^−1^. Pulling simulations were performed by applying a harmonic biasing force of 10 kJ mol^−1^ Å^−2^ between the COM of the peptide and the COM of the bilayer. Then, from the steered MD simulation trajectory, selected every 1 Å, 100 configurations were generated along the z-axis direction (reaction coordinates), and used as starting points for umbrella sampling simulations. Subsequently, each window was simulated for 50 ns with the pull “distance” coordinate geometry. Weighted histogram analysis (WHAM) of the last 10 ns of umbrella sampling simulations was used to construct the PMF profiles [27].

## 3. Results

### 3.1. CD Studies of Peptide-ANM Liposomes Interactions

In aqueous solutions, unbound RRCPPs generally adopt an unordered conformation [28,29,30]. In rare cases, they may form a polyproline type II helical conformation [31,32]. This is mostly related to a strong repulsive interaction between the quinidine groups of the arginine residues. Modeling the Tat’s arginine-rich motif (ARM) structure to achieve the most stable helical conformation has favored the development of the PTD4 peptide, in which the replacement of particular lysine and arginine by alanine residues (Lys^50,51^,Arg^52,55,57^→Ala) resulted in a Tat(49–57)-NH_2_ analog with an enhanced membrane transduction potential [33].

Our previous data showed that both Tat(49–57)-NH_2_ and PTD4 were neuroprotective in an in vitro model of acute ischemic stroke [13]. CD data and MD simulations showed that replacement of particular lysine and arginine with alanine residues enhances the ability of the PTD4 peptide to adopt a helical conformation in a hydrophobic environment [13].

RRCPPs have been shown to exhibit intrinsic neuroprotective properties which correlated with their ability to cross the cellular membrane [6,12,13,34]. The interaction of the peptide with the membrane is the first step in the neuroprotective mechanism. We also observed that the conformational properties of the peptides and their cell membrane-penetrating ability may play a significant role in their neuroprotection [6]. To characterize the peptide–cell membrane interaction, we performed CD spectroscopy analysis. ANM was used to simulate the native neural cellular membrane [14,15].

**Figure 1:**
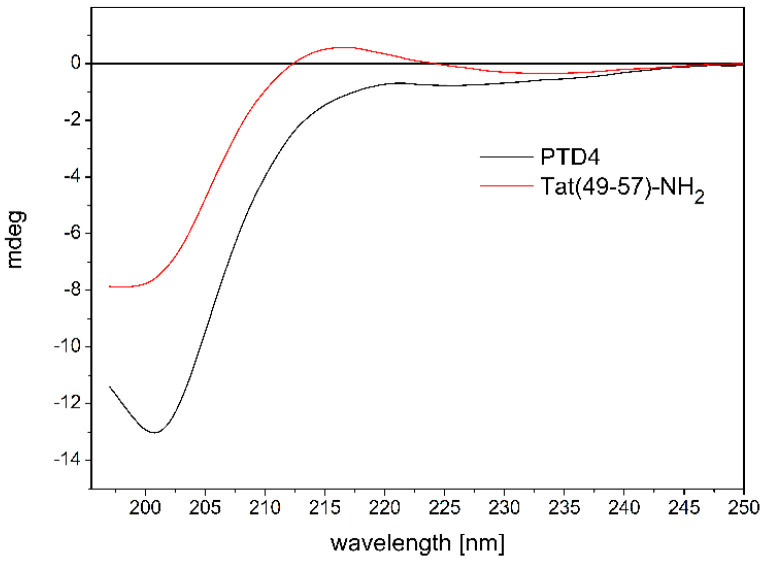
CD spectra showing the influence of Ala-scan substitution (Lys^50,51^,Arg^52,55,57^→Ala) on the conformational properties of Tat(49–57)-NH_2_.

To analyze the impact of conformational properties of the peptides on their potential to interact with ANM liposomes, we first investigated the behavior of the peptides in an aqueous environment. The influence of Ala-scan substitution (Lys^50,51^,Arg^52,55,57^→Ala) on the conformational properties of Tat(49–57)-NH_2_ and PTD4 peptides was evaluated by CD spectroscopy (Figure 1).

The CD spectrum of unbound Tat(49–57)-NH_2_, which was recorded in 10 mM PBS (pH 7.0) at 37 °C (Figure 1), showed a broad negative minimum at *λ* ≈ 200 nm and a shallow maximum at *λ* ≈ 215 nm. The shape of the CD curve was typical for a random coil conformation of the peptide backbone. However, it should be mentioned that a rare helical-type conformation of polyproline type II also gives a similar signal in the CD spectra. Our previous structural data on Tat(49–57)-NH_2_ indicated that a mixture of both conformations is present in the aqueous solution [13].

In contrast, the CD spectra of PTD4 showed a broad minimum at *λ* ≈ 200 nm, which moved slightly toward longer wavelengths, compared to the Tat peptide with an additional shallow and wide maximum of negative ellipticity at *λ* ≈ 220 nm (Figure 1). This indicates a random coil conformation with a slight trace of the formation of ordered structures around the maximum extremum. The last one may be related to the effect of the substitution of lysine and arginine residues bearing positively charged amino and guanidinium groups by nonpolar alanine residues. This substitution favors the helical conformation of the peptide chain. Thus, the CD curves showed a subtle influence of the Ala-scan substitution on the conformational properties of the peptides.

Since peptide conformation-based interactions with the neuronal cell membrane may contribute to the neuroprotective properties of RRCPPs, we performed CD spectroscopy to investigate the conformational behavior of Tat(49–57)-NH_2_ and PTD4 peptides during their interaction with ANM liposomes. The composition of liposomes mimicked that of lipids on the natural neuronal membrane [14,15,35].

**Figure 2:**
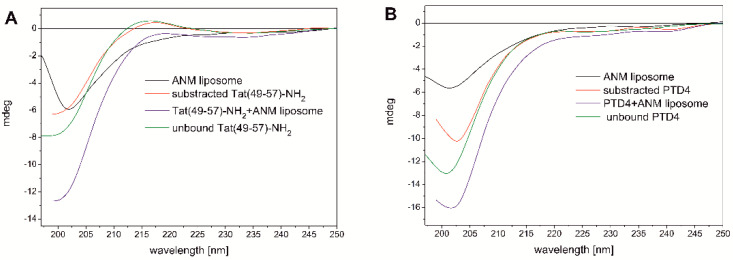
CD spectra showing the conformational behavior of Tat(49–57)-NH_2_ (**A**) and PTD4 (**B**) peptides during their interaction with ANM liposomes.

The liposome solution (1.25 mM) resulted in significant negative ellipticity at *λ* ≈ 200 nm, the intensity of which was comparable to the signal of Tat(49–57)-NH_2_ and much less compared to the PTD4 peptide (Figure 2A,B). The CD curve of the ANM liposome closely resembled the CD curve typical for the unordered peptide conformation. Such a CD curve has been reported previously for liposomes comprising the cell membrane (however not the neuronal type) from lipids [36]. The CD curve minimum of the Tat(49–57)-NH_2_ peptide and the ANM liposome was located almost at the same location at *λ* ≈ 200 nm (Figure 2A). However, the minimum of PTD4 was slightly red-shifted compared to that of the liposome(Figure 2B). The decreasing in the intensity of the CD signal at about 200 nm in combination with its redshift effect is probably a symptom of the formation of ordered structures during the interaction of the PTD4 peptide with the ANM liposomes.

The ellipticity signal of the ANM liposomes originated from the chiral structural elements of individual lipids used for their preparation [37].

Dissolving the peptides in the liposome-containing solution led to a significant increase in total ellipticity at *λ* ≈ 220 nm, as well as in the long-wavelength part of the spectrum (Figure 2A,B). This indicates a synergistic effect of the overlapping elliptical signals of the peptide and liposome as well. For Tat(49–57)-NH_2_, this synergy led to the disappearance of the shallow positive maximum that was seen on the peptide’s CD curve. The interactions of the PTD4 peptide with the liposomes also caused the minimum at *λ* ≈ 200 nm to slightly shift toward longer wavelengths in relation to the unbound peptide and the liposomes (Figure 2B).

A comparison of the CD curves recorded for the unbound Tat(49–57)-NH_2_ peptide with that showing conformational changes of the peptide due to its interaction with the liposomes (the CD signal of the peptide subtracted from the total signal of the peptide–liposome solution) revealed that the peptide underwent relatively small conformational changes (Figure 2A). Only a slight decrease in elliptical intensity was observed in the region of the minimum at *λ* ≈ 200 nm and the maximum at *λ* ≈ 215 nm.

Similar changes were also observed in the case of the PTD4 peptide (Figure 2B). The decrease of the minimum at *λ* ≈ 200 nm was more intense compared to the Tat peptide, and virtually no changes were noted in the long-wavelength part of the CD spectrum. Based on a comparison of the CD curve of the unbound PTD4 peptide with that obtained as a result of subtracting the peptide signal from the solution containing the liposome–peptide mixture, it was found that the most noticeable difference from the Tat peptide was a redshift of the negative minimum at about 200 nm

### 3.2. CG MD Simulations of Spontaneous Peptide–Membrane Interactions

**Figure 3:**
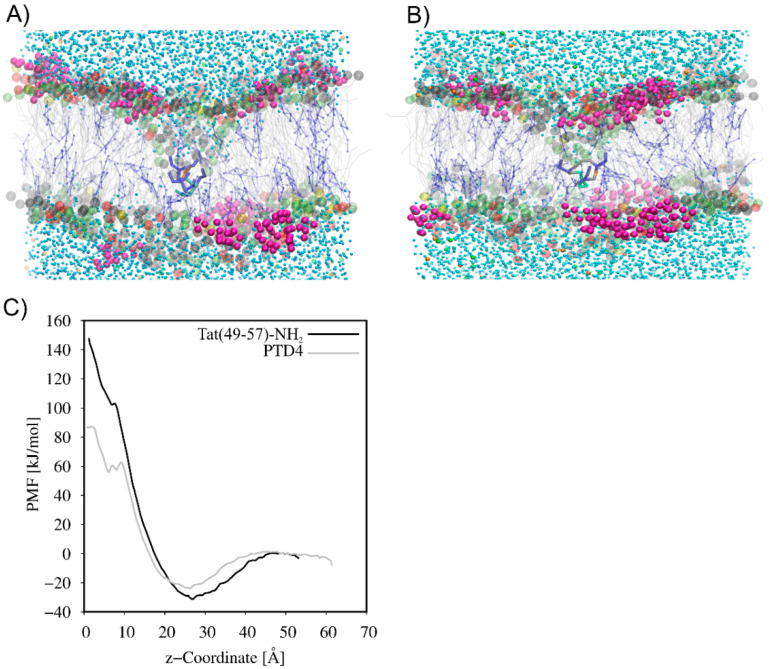
Formation of hydrophilic pores in pulling CG MD simulations of (**A**) Tat(49–57)-NH_2_ and (**B**) PTD4 across the neuronal membrane model. (**C**) Comparison of potential mean of force (PMF) between Tat(49–57)-NH_2_ and PTD4. The headgroups of POPC, POPE, POPS and DPSM are colored gray, lime, yellow and red, respectively. The sugar part of DPG1 is in magenta, while lipid acyl chains are presented in grey. CHOL is presented in blue as CPK model. The amino acid residues in licorice representation are colored as follows: Arg-blue, Lys-cyan, Gln-orange, Tyr-green and Ala-grey.

The CG MD simulations were carried out to understand the mechanism of Tat(49–57)-NH_2_ and PTD4 interactions with the neuronal lipid bilayer. In the beginning, systems with a single peptide molecule were studied. As expected, the peptides, driven by electrostatic interactions, adsorbed quickly on the membrane surface. However, neither of the peptides penetrated the membrane, and both remained attracted to the membrane surface at the end of 10-µs CG-MD simulations (Supplementary Figure S1). Therefore, to induce the movement of peptides across the neuronal lipid bilayer, pulling CG MD simulations were used. As seen in Figure 3, the translocation of peptides was accompanied by the formation of hydrophilic pores and the exchange of water molecules on both sides of the membrane. The PMF curves (Figure 3C) showed the local minimum as the peptides approached the membrane surface. This finding indicates that peptide adsorption on the membrane surface is energetically favorable. A high free energy barrier of 180 kJ mol^−1^ and 110 kJ mol^−1^ was observed for Tat(49–57)-NH_2_ and PTD4, respectively, which prevented the penetration of the peptides into the lipid bilayer. The high energy barrier was formed as a result of strong electrostatic attraction between the positively charged peptide and the negatively charged membrane surface, as well as unfavorable interactions between the hydrophilic peptide and hydrophobic core of the membrane. Thus, the PMF analysis strongly supported the observation that a single Tat(49–57)-NH_2_ and PTD4 peptide cannot spontaneously cross the lipid membrane.

**Figure 4:**
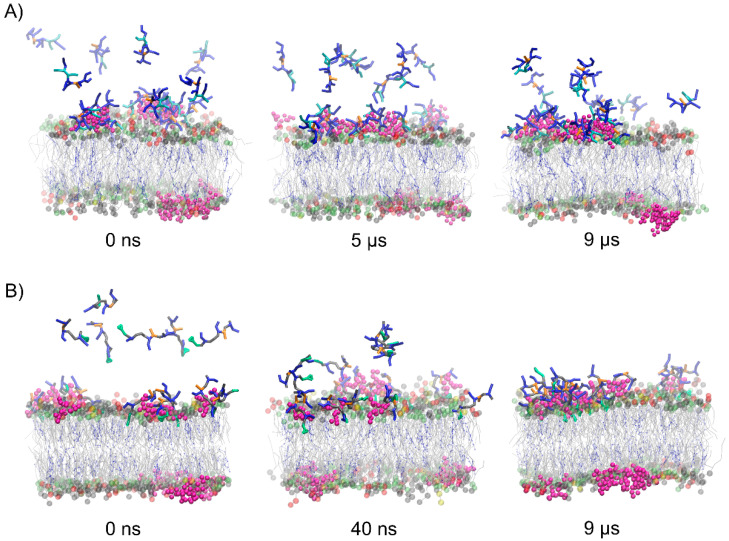
Representative snapshots from the POPC:POPE:POPS:DPG1:DPSM:CHOL binding CG MD simulations for multipeptide systems with Tat(49–57)-NH_2_ (**A**) and PTD4 (**B**). The headgroups of POPC, POPE, POPS, and DPSM are colored gray, lime, yellow, and red, respectively. The sugar part of DPG1 is in magenta, while lipid acyl chains are presented in gray. CHOL is presented in blue as CPK model.

**Figure 5:**
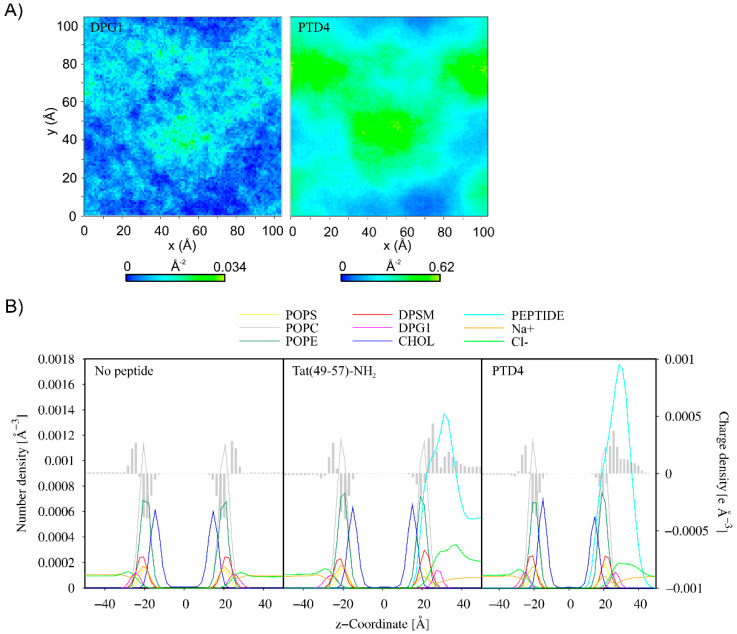
(**A**) 2D density map of DPG1 lipids and PTD4 in the upper leaflet of the membrane. The grid spacing was set to 1 Å. The last 1 µs of 10-µs CG MD simulations was considered for the analysis. (**B**) Partial density and charge density profiles averaged over the last 1 µs of MD CG simulations of the systems without peptide and with multiple Tat(49–57)-NH_2_ and PTD4 molecules.

To evaluate the effect of concentration of the peptides on their membrane-penetrating efficiency, CG MD simulations were performed for multipeptide systems. In the early stages of simulations, the peptide molecules were quickly attracted to the membrane surface. However, each membrane-bound peptide molecule gradually neutralized the surface charge of the membrane, impairing the attraction of subsequent positively charged peptide molecules. In the case of Tat(49–57)-NH_2_ peptide with a greater overall positive charge, we noted that about half of the molecules remained in an unbound aqueous state at the end of simulations (Figure 4). Interestingly, less polar PTD4 self-assembled before it reached the membrane surface, with the central alanine triblock hidden inside the oligomers that were being formed. A close inspection analysis of peptide–membrane interactions revealed that DPG1 lipids played a major role in peptide binding. These lipids formed clusters within the neuronal membrane, which were the preferable site for peptide binding (Figure 5). The positively charged peptides covering the membrane surface induced changes in its charge density, and the distribution of sodium and chloride ions at the outer lipid–water interface. Unfortunately, membrane penetration was not evident with increasing peptide concentration.

## 4. Discussion

CD spectroscopy results indicated that both peptides assume a random coil conformation in the unbound aqueous state and their ability to accept ordered (helical) structures is relatively low. This shows that the Ala-scan procedure does not significantly affect the conformational properties of RRCPPs, and that the positively charged side chains of lysine and arginine residues play a key role in the adoption of a disordered structure by the peptides.

Interaction with the ANM liposome resulted in slight conformational changes in the peptide chain in both Tat(49–57)-NH_2_ and PTD4. These were manifested mainly as a reduction in the minimum intensity of the CD curve at *λ* ≈ 200 nm. For the PTD4 peptide, the reduction in minimum intensity was accompanied by a red-shift effect, while in the case of the Tat(49–57)-NH_2_ peptide a slight decrease in the intensity of the shallow maximum at *λ* ≈ 215 nm was additionally observed. This shows that the interaction of RRCPPs with the hydrophobic environment of the neuronal membrane and their potential penetration induce slight conformational changes in these peptides. However, the intensity of these changes is not high, and the exact nature of change could not be easily determined based on the effects observed in CD analysis. The reduction of the minimum intensity at *λ* ≈ 200 nm, which is characteristic of the disordered conformation, may suggest a slight shift of the conformational equilibrium toward ordered, probably helical structures. Previous conformational studies have shown that basic peptides such as Tat can form helical structures (like polyproline type II) in a hydrophobic environment or when bound by a ligand [38,39].

The membrane-penetrating efficiency of peptides depends on the interactions between the peptide and the lipid headgroups at the bilayer–water interface [39]. Before the peptide penetrates the membrane, it is essential to disrupt the strong electrostatic attraction between the peptide and the membrane surface. The results of the CG MD simulations confirmed that DPG1-enriched rafts are the major binding site for Tat(49–57)-NH_2_ and PTD4. The peptides get rapidly attracted to the sugar part of the DPG1 and remain in the bound state at the end of the simulations. This strong interaction may be limiting the spontaneous membrane penetration of peptides even at increasing concentrations.

The ability to interact with the neuron membrane and its subsequent penetration by CPPs seems to be a key factor determining their neuroprotective effect. Earlier data showed that the ability of peptides to penetrate the cell membrane correlates with their helicity and neuroprotective ability [13]. Our research indicated that Tat(49–57)-NH_2_ and PTD4 peptides interacted with the ANM liposomes which mimicked the membrane of a neuron. The CD spectra showed an intense elliptical signal from the ANM liposomes. The shape of the CD curve resembled that obtained for peptides with a random coil conformation. Its elliptical intensity was comparable to or less than that observed for Tat and PTD4 peptides. As a result, we investigated the ANM elliptical effect of liposomes interacting with peptides. The interactions caused relatively small conformational changes of peptides together with the shift of the polar water environment to the hydrophobic ANM environment of the liposome. However, these changes were so small to explain whether they lead to an ordered structure of the peptide chain and the formation of helical structures, which would be expected, especially in the case of the PTD4 peptide. The similar behavior of both peptides observed in CD studies correlates with their similar neuroprotective properties. Our previous results showed that both the neuroprotective response profile to stressors included in the in vitro model and the magnitude of the response for both peptides were similar [13]. The similar behavior of peptides during their interaction with the ANM liposomes does not confirm the previous reports suggesting that the PTD4 peptide had a 30-fold higher ability to penetrate the cell membrane compared to the Tat(49–57)-NH_2_ peptide due to its greater tendency to penetrate the cell membrane and adopt helical structures.

In conclusion, although the penetrating ability of arginine-rich peptides (such as Tat (49–57)-NH_2_ and its analog PTD4) has been well established, our studies did not show a direct correlation between the penetration efficiency and conformational changes occurring in peptides during interaction with the ANM liposomes (CD studies) and translocation across a model cell membrane (CG MD simulations). The conformational changes observed in peptides during their interaction with the ANM liposomes in CD studies were so small as to prove a correlation between the structural changes in peptides (e.g., a tendency to form a helical structure) and the ability of peptides to penetrate cell membranes. Likewise, both the studied peptides did not show any move across the cell membrane during CG MD simulations of peptide–membrane interactions. These simulations showed that the high free energy barrier (supported by the PMF analysis) of both peptides (higher for Tat(49–57)-NH_2_ than PTD4) prevents their penetration into the lipid bilayer. The free energy profile (i.e., free energy barrier and PMF value) is commonly used to explain the translocation of different CPPs across the lipid membranes and compare the penetrating efficiency of CPPs with a similar charge [39]. As suggested, the free energy barrier is inversely proportional to the penetration efficiency of CPPs, as reported experimentally, and may be useful to distinguish peptides characterized by different penetration efficiencies. Thus, the lower free energy barrier observed for PTD4 may explain its higher penetration efficiency compared to Tat(49–57)-NH_2_.

The main problem in the simulations carried out is the lack of the peptide penetration effect. Due to the high positive charge of the CPP peptides, electrode interactions are favored, the consequence of which is their adsorption on the membrane surface. The high energy of these interactions, combined with the unfavorable interactions of the hydrophilic peptide with the hydrophobic interior of the membrane, prevents the CPP peptide from crossing the membrane barrier. Choe [40] obtained a similar result, but it was obtained with a different MD-weighted ensemble (WE) method. In his simulations, the Tat peptide also penetrated the cell membrane (it had a different composition than in our research), but it did not spontaneously exceed it. It is known from previous studies that the mechanism of penetration of the cell membrane by CPPs is very sensitive to environmental conditions [1,41]. Even for a specific peptide, different penetration mechanisms were observed depending on the conditions of the experiment [41,42]. It is therefore possible that the previously used MD simulation algorithms for studying the interactions of the membrane with peptide ligands are too simplified or do not contain some essential element that would allow the penetration of the cell membrane. It is known that endocytosis is a common membrane mechanism by CPPs, both free and cargo-carrying [42]. This process depends on many factors, such as the presence of sulfonated proteoglycans or membrane receptors. The presence of these factors was not taken into account by our model of the neural membrane used in the CD and CG MD studies. This is just one example showing that the simplified membrane model used in our and previous literature studies may not simulate the key membrane features necessary for the translocation process of CPPs.

The presented research results are one of the first attempts to correlate the neuroprotective activity of CPPs with their interaction with the neuronal membrane. For this reason, we decided to move in close to physiological conditions. It is known, however, that during ischemic stroke, neurons experience many pathophysiological changes, such as lowering the pH and increasing the concentration of calcium ions [43,44]. These changes may also concern the composition and properties of the neuronal membrane. For this reason, it should be taken into account that under pathogenic conditions the interaction of neuroprotective CPPs with the neuronal membrane may differ significantly from those under physiological conditions. In further experiments, we intend to investigate whether such differences are visible and significant for the interaction of CPPs with the neuronal membrane.

In summary, the presented research results show some details related to the interaction of selected RRCPPs with the neuronal cell membrane. Unfortunately, we have not been able to get a complete picture showing the mechanism of peptide penetration of the neuronal membrane. This clearly shows the need for further research to explain the phenomenon of the neuroprotective activity of RRCPPs and its relationship with the penetration of the neuron membrane.

## Data Availability

The datasets used and/or analyzed during the current study are available from the corresponding author on reasonable request.

## Supplementary Materials

The following supporting information can be downloaded at: https://www.mdpi.com/article/10.3390/cells11101638/s1, Figure S1: The final snapshots from the POPC:POPE:POPS:DPG1:DPSM:CHOL binding CG MD simulations for systems with a single molecule of Tat(49-57)-NH_2_ (A) and PTD4 (B); Table S1: Composition of the bilayer systems.

## Abbreviations

AIS	acute ischemic stroke
ANM	artificial neuronal membrane
ARM	arginine rich motif
CD	circular dichroism
CG MD	coarse-grained molecular dynamics
CHOL	cholesterol
CPP	cell-penetrating peptide
DPG1	monosialotetrahexosylganglioside
DPSM	sphingomyelin
HIV	human immunodeficiency virus
HPLC	high-performance liquid chromatography
L/P	lipid-peptide molar ratio
PMF	potential mean of force
POPC	1-palmitoyl-2-oleoyl-sn-glycero-3-phosphocholine
POPE	1-palmitoyl-2-oleoyl-sn-glycero-3-phosphoethanolamine
POPS	1-palmitoyl-2-oleoyl-sn-glycero-3-phospho-L-serine
PTD	protein transduction domain
RRCPP	arginine-rich cell-penetrating peptide
Tat	transactivator of transcription

## References

[1] Ruczyński J., Wierzbicki P.M., Kogut-Wierzbicka M., Mucha P., Siedlecka-Kroplewska K., Rekowski P. (2014). Cell-penetrating peptides as a promising tool for delivery of various molecules into the cells. Folia Histochem. Cytobiol..

[2] Rusiecka I., Ruczyński J., Kozłowska A., Backtrog E., Mucha P., Kocić I., Rekowski P. (2019). TP10-dopamine conjugate as a potential therapeutic agent in the reatment of Parkinson’s disease. Bioconjug. Chem..

[3] Durzyńska J., Przysiecka Ł., Nawrot R., Barylski J., Nowicki G., Warowicka A., Musidlak O., Goździcka-Józefiak A. (2015). Viral and other cell-penetrating peptides as vectors of therapeutic agents in medicine. J. Pharmacol. Exp. Ther..

[4] Gallo M., Defaus S., Andreu D. (2019). 1988-2018: Thirty years of drug smuggling at the nano scale. Challenges and opportunities of cell-penetrating peptides in biomedical research. Arch. Biochem. Biophys..

[5] Trabulo S., Cardoso A.L., Mano M., De Lima M.C. (2010). Cell-Penetrating Peptides-Mechanisms of Cellular Uptake and Generation of Delivery Systems. Pharmaceuticals.

[6] Meloni B.P., Mastaglia F.L., Knuckey N.W. (2020). Cationic Arginine-Rich Peptides (CARPs): A Novel Class of Neuroprotective Agents with a Multimodal Mechanism of Action. Front. Neurol..

[7] Mendelson S.J., Prabhakaran S. (2021). Diagnosis and Management of Transient Ischemic Attack and Acute Ischemic Stroke: A Review. JAMA.

[8] Marto J.P., Strambo D., Livio F., Michel P. (2021). Drugs Associated with Ischemic Stroke: A Review for Clinicians. Stroke.

[9] Towfighi A., Saver J.L. (2011). Stroke declines from third to fourth leading cause of death in the United States: Historical perspective and challenges ahead. Stroke.

[10] National Institute of Neurological Disorders and Stroke rt-PA Stroke Study Group (1995). Tissue plasminogen activator for acute ischemic stroke. N. Engl. J. Med..

[11] Meloni B.P., Brookes L.M., Clark V.W., Cross J.L., Edwards A.B., Anderton R.S., Hopkins R.M., Hoffmann K., Knuckey N.W. (2015). Poly-arginine and arginine-rich peptides are neuroprotective in stroke models. J. Cereb. Blood Flow Metab..

[12] Meloni B.P., Craig A.J., Milech N., Hopkins R.M., Watt P.M., Knuckey N.W. (2014). The neuroprotective efficacy of cell-penetrating peptides TAT, penetratin, Arg-9, and Pep-1 in glutamic acid, kainic acid, and in vitro ischemia injury models using primary cortical neuronal cultures. Cell Mol. Neurobiol..

[13] Mazuryk J., Puchalska I., Koziński K., Ślusarz M.J., Ruczyński J., Rekowski P., Rogujski P., Płatek R., Wiśniewska M.B., Piotrowski A. (2021). PTD4 Peptide Increases Neural Viability in an In Vitro Model of Acute Ischemic Stroke. Int. J. Mol. Sci..

[14] Fatafta H., Khaled M., Owen M.C., Sayyed-Ahmad A., Strodel B. (2021). Amyloid-β peptide dimers undergo a random coil to β-sheet transition in the aqueous phase but not at the neuronal membrane. Proc. Natl. Acad. Sci. USA.

[15] Bera S., Gayen N., Mohid S.A., Bhattacharyya D., Krishnamoorthy J., Sarkar D., Choi J., Sahoo N., Mandal A.K., Lee D. (2020). Comparison of Synthetic Neuronal Model Membrane Mimics in Amyloid Aggregation at Atomic Resolution. ACS Chem. Neurosci..

[16] Mucha P., Szyk A., Rekowski P., Barciszewski J. (2002). Structural requirements for conserved Arg52 residue for interaction of the human immunodeficiency virus type 1 trans-activation responsive element with trans-activator of transcription protein (49–57). Capillary electrophoresis mobility shift assay. J. Chromatogr. A.

[17] Wojciechowska M., Ruczynski J., Rekowski P., Alenowicz M., Mucha P., Pieszko M., Miszka A., Dobkowski M., Bluijssen H. (2014). Synthesis and Hybridization Studies of a New CPP-PNA Conjugate as a Potential Therapeutic Agent in Atherosclerosis Treatment. Protein Pept. Lett..

[18] Periole X., Marrink S.J. (2013). The Martini coarse-grained force field. Methods Mol. Biol..

[19] Marrink S.J., Risselada H.J., Yefimov S., Tieleman D.P., de Vries A.H. (2007). The MARTINI force field: Coarse grained model for biomolecular simulations. J. Phys. Chem. B.

[20] Hess B., Kutzner C., Van Der Spoel D., Lindahl E. (2008). GROMACS 4: Algorithms for highly efficient, load-balanced, and scalable molecular simulation. J. Chem. Theory Comput..

[21] Lee J., Lee L., Cheng X., Swails J.M., Yeom M.S., Eastman P.K., Lemkul J.A., Wei S., Buckner J., Jeong J.C. (2016). CHARMM-GUI Input Generator for NAMD, GROMACS, AMBER, OpenMM, and CHARMM/OpenMM Simulations Using the CHARMM36 Additive Force Field. J. Chem. Theory Comput..

[22] Jo S., Kim T., Iyer V.G., Im W. (2008). CHARMM-GUI: A web-based graphical user interface for CHARMM (in eng). J. Comput. Chem..

[23] Brooks B.R., Brooks C.L., MacKerell A.D., Nilsson L., Petrella R.J., Roux B., Won Y., Archontis G., Bartels C., Boresch S. (2009). CHARMM: The biomolecular simulation program. J. Comput. Chem..

[24] Wu E.L., Cheng X., Jo S., Rui H., Song K.C., Dávila-Contreras E.M., Qi Y., Lee J., Monje-Galvan V., Venable R.M. (2014). CHARMM-GUI membrane builder toward realistic biological membrane simulations. J. Comput. Chem..

[25] Winger M., Trzesniak D., Baron R., van Gunsteren W.F. (2009). On using a too large integration time step in molecular dynamics simulations of coarse-grained molecular models. Phys. Chem. Chem. Phys..

[26] Lemkul J.A., Bevan D.R. (2010). Assessing the stability of Alzheimer’s amyloid protofibrils using molecular dynamic. J. Phys. Chem. B.

[27] Kumar S., Rosenberg J.M., Bouzida D., Swendsen R.H., Kollman P.A. (1992). The weighted histogram analysis method for free-energy calculations on biomolecules. I. The method. J. Comput. Chem..

[28] Kalafatovic D., Giralt E. (2017). Cell-Penetrating Peptides: Design Strategies beyond Primary Structure and Amphipathicity. Molecules.

[29] Szyk A., Mucha P., Rekowski P., Giel-Pietraszuk M., Barciszewski J. (1999). Synthesis and circular dichroism studies of HIV-1 Tat arginine rich domain analogues substituted in Arg 52 position. Pol. J. Chem..

[30] Oba M., Nagano Y., Kato T., Tanaka M. (2019). Secondary structures and cell-penetrating abilities of arginine-rich peptide foldamers. Sci. Rep..

[31] Ruzza P., Calderan A., Guiotto A., Osler A., Borin G. (2004). Tat cell-penetrating peptide has the characteristics of a poly(proline) II helix in aqueous solution and in SDS micelles. J. Pept. Sci..

[32] Lam S.L., Hsu V.L. (2003). NMR identification of left-handed polyproline type II helices. Biopolymers.

[33] Ho A., Schwarze S.R., Mermelstain S.J., Waksman G., Dowdy S.F. (2001). Synthetic Protein Transduction Domains: Enhanced Transduction Potential in Vitro and in Vivo. Cancer Res..

[34] Vaslin A., Rummel C., Clarke P.G. (2009). Unconjugated TAT carrier peptide protects against excitotoxicity. Neurotox. Res..

[35] Andrade S., Loureiro J.A., Pereira M.C. (2021). Vitamin B12 Inhibits Aβ Fibrillation and Disaggregates Preformed Fibrils in the Presence of Synthetic Neuronal Membranes. ACS Chem. Neurosci..

[36] Mizuno S., Sasai H., Kume A., Takahashi D., Satoh M., Kado S., Sakane F. (2017). Dioleoyl-phosphatidic acid selectively binds to α-synuclein and strongly induces its aggregation. FEBS Lett..

[37] Long K.S., Crothers D.M. (1999). Characterization of the solution conformations of unbound and Tat peptide-bound forms of HIV-1 TAR RNA. Biochemistry.

[38] Aboul-ela F., Karn J., Varani G. (1995). The structure of the human immunodeficiency virus type-1 TAR RNA reveals principles of RNA recognition by Tat protein. J. Mol. Biol..

[39] Her Choong F., Keat Yap B. (2021). Cell-penetrating peptides: Correlation between peptide-lipid interaction and penetration efficiency. ChemPhysChem.

[40] Choe S. (2021). Free Energy Analyses of Cell-Penetrating Peptides Using the Weighted Ensemble Method. Membranes.

[41] Ruseska I., Zimmer A. (2020). Internalization mechanisms of cell-penetrating peptides. Beilstein J. Nanotechnol..

[42] Liu B.R., Chiou S.-H., Huang Y.-W., Lee H.-J. (2022). Bio-Membrane Internalization Mechanisms of Arginine-Rich Cell-Penetrating Peptides in Various Species. Membranes.

[43] Meloni B.P., Milani D., Edwards A.B., Anderton R.S., O’Hare Doig R.L., Fitzgerald M., Palmer T.N., Knuckey N.W. (2015). Neuroprotective peptides fused to arginine-rich cell penetrating peptides: Neuroprotective mechanism likely mediated by peptide endocytic properties. Pharmacol. Ther..

[44] Woodruff T.M., Thundyil J., Tang S.-C., Sobey C.G., Taylor S.M., Arumugam T.V. (2011). Pathophysiology, treatment, and animal and cellular models of human ischemic stroke. Mol. Neurodegener..

