# Supplementary material for: Interaction of Arginine-Rich Cell-Penetrating Peptides with an Artificial Neuronal Membrane
Source: Cells. 2022 May 13;11(10):1638. doi: 10.3390/cells11101638 (PMC9139471; doi:10.3390/cells11101638)
Supplement: Supplementary file 1 [file cells-11-01638-s001.zip › cells-1681080-supplementary.pdf]

cells

MDPI

Supplementary Materials

Table S1. Composition of the bilayer systems.

Model	Lipid types		Ion types		Peptide	Water	Initial system size [x,y,x; Å]	Simulation time [μs]
	Outer	Inner	Na^+	Cl^-				
No pep-<br>tide	76 POPC<br>48 POPE<br>10 POPS<br>40 CHOL<br>18 DPSM<br>8 DPG1	76 POPC<br>48 POPE<br>10 POPS<br>40 CHOL<br>18 DPSM<br>8 DPG1	284	248	-	22112	117,117,200	5
TAT_1	76 POPC<br>48 POPE<br>10 POPS<br>40 CHOL<br>18 DPSM<br>8 DPG1	76 POPC<br>48 POPE<br>10 POPS<br>40 CHOL<br>18 DPSM<br>8 DPG1	213	186	1	16254	117,117,150	10
TAT_15	76 POPC<br>48 POPE<br>10 POPS<br>40 CHOL<br>18 DPSM<br>8 DPG1	76 POPC<br>48 POPE<br>10 POPS<br>40 CHOL<br>18 DPSM<br>8 DPG1	248	302<br>347*	10<br>15*	21935<br>21853*	117,117,200	1<br>9
PTD4_1	76 POPC<br>48 POPE<br>10 POPS<br>40 CHOL<br>18 DPSM<br>8 DPG1	76 POPC<br>48 POPE<br>10 POPS<br>40 CHOL<br>18 DPSM<br>8 DPG1	218	186	1	16301	117,117,150	10
PTD4_15	76 POPC<br>48 POPE<br>10 POPS<br>40 CHOL<br>18 DPSM<br>8 DPG1	76 POPC<br>48 POPE<br>10 POPS<br>40 CHOL<br>18 DPSM<br>8 DPG1	248	252<br>272*	10<br>15*	22002<br>21949*	117,117,200	1<br><br>9

* Number of peptide molecules, chloride ion and water molecules after adding additional five peptide molecules to the system after 1 μs of the CG MD simulation. The CG MD simulations were then continued for another 9 μs.

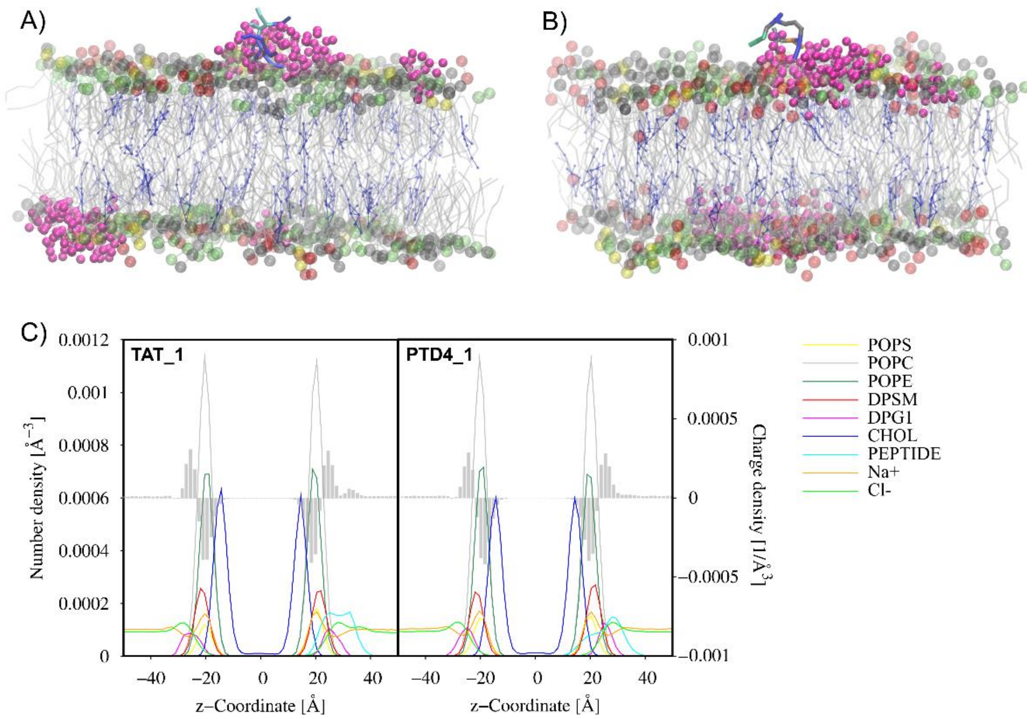

Figure S1. The final snapshots from the POPC:POPE:POPS:DPG1:DPSM:CHOL binding CG MD simulations for systems with a single molecule of Tat(49-57)-NH_2 (A) and PTD4 (B). The headgroups of POPC, POPE, POPS and DPSM are colored gray, lime, yellow and red, respectively. The sugar part of DPG1 is in magenta, while lipid acyl chains are presented in grey. CHOL is presented in blue as CPK model. (C) Partial density and charge density profiles averaged over the last 1 μs of MD CG simulations of the systems with a single peptide molecule.
